# Utilization of cardiac graft with single coronary artery for orthotopic heart transplantation

**DOI:** 10.1186/s13019-022-02035-x

**Published:** 2022-11-18

**Authors:** Nicholas R. Hess, Mary E. Keebler, Carly A. Fabrizio, David J. Kaczorowski

**Affiliations:** 1 Division of Cardiac Surgery, Department of Cardiothoracic Surgery, University of Pittsburgh School of Medicine, 200 Lothrop Street, Suite C-700, Pittsburgh, PA 15213 USA; 2 University of Pittsburgh Medical Center Heart and Vascular Institute, Pittsburgh, PA USA; 3 Heart and Vascular Institute, Christiana Health Care System, Newark, DE USA

**Keywords:** Orthotopic heart transplantation, Anomalous coronary artery, Single coronary artery

## Abstract

**Background:**

Anomalous coronary arteries arise in a small subset of the population, with each configuration conveying a varying degree of long-term risk. The utilization of cardiac grafts with these anomalies have not been well described.

**Case presentation:**

An anomalous single coronary artery with the left main coronary artery arising from the right coronary ostium was discovered in a 40-year old male evaluated for cardiac donation. After evaluation, this heart was successfully procured and utilized for orthotopic heart transplantation.

**Conclusion:**

In this report, we demonstrate that in select cases, a cardiac graft with single coronary artery anatomy can be successfully procured and transplanted with excellent outcomes.

**Supplementary Information:**

The online version contains supplementary material available at 10.1186/s13019-022-02035-x.

## Background

Coronary artery anomalies are uncommon, arising in approximately 0.1–5% of the population, depending on series [1]. While some variations may be associated with increased risk of ischemic events or sudden death, others may be benign findings which do not pose additional risk nor warrant treatment. Even less described is the usage of donor hearts with anomalous coronary anatomy for transplantation. In this article, we present a case of successful utilization of a donor graft with a single coronary artery for transplantation.

## Case presentation

This study was approved by our institutional review board (MOD18120143-003, approved 3/9/2020), and written consent was obtained.

A 60-year-old male with history of nonischemic cardiomyopathy and left ventricular ejection fraction of 20% was admitted to the hospital with recurrent ventricular tachycardia. He had undergone three unsuccessful ablation procedures and had also failed multiple outpatient anti-arrhythmic pharmacotherapies. While inpatient, he was maintained on a continuous intravenous infusion of lidocaine. Initially, he was listed as Status 3E, and remained inpatient awaiting a heart offer for over four months. Eventually, he was upgraded to Status 2E due to increased burden of ventricular tachycardia. On post-upgrade day 16, he received a donor offer.

**Fig. 1:**
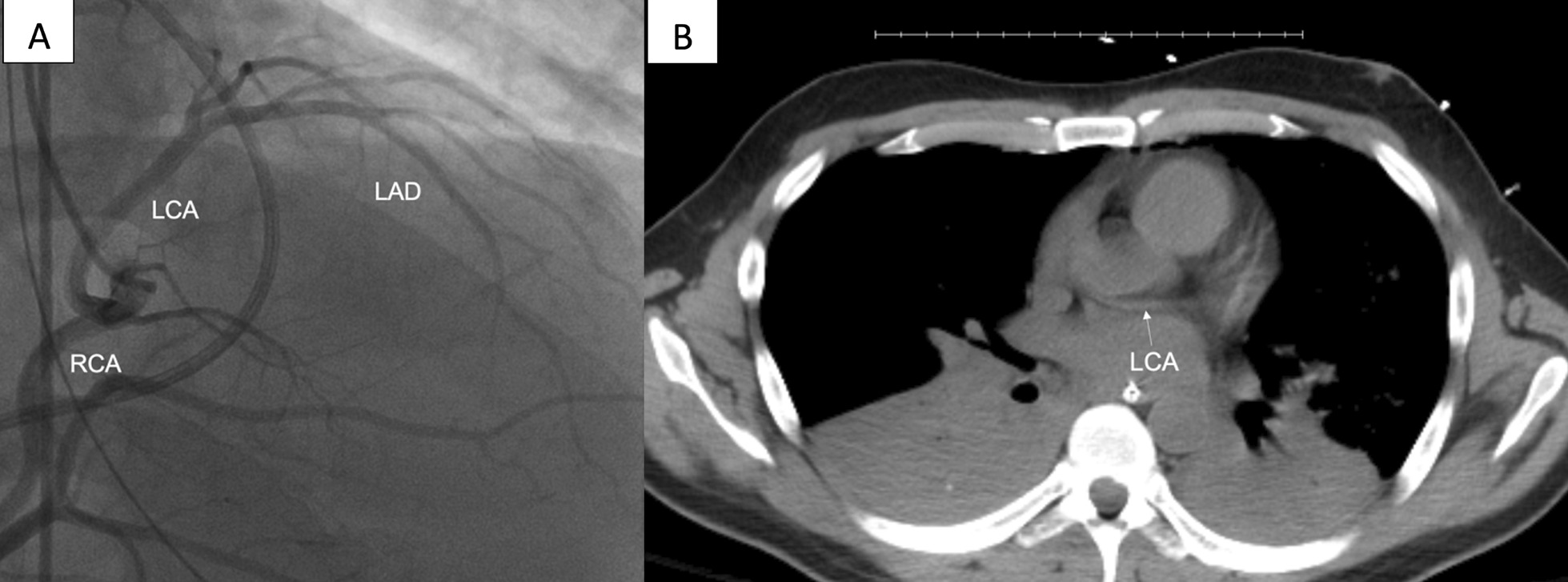
Evaluation of donor cardiac graft included **A** left heart angiogram demonstrating both left and right coronary arteries arising from single ostium within the right sinus of Valsalva and **B** chest computed tomography revealing retro-aortic course of the left coronary artery. *LAD* left anterior descending, *LCA* left coronary artery, *RCA* right coronary artery

The donor was a 40-year-old male who underwent brain death pronouncement after sustaining blunt head trauma. On echocardiography, the heart had good biventricular function. Due to the donor’s age, left coronary catheterization was performed, which revealed an anomalous origin of the left coronary artery (LCA). The LCA and right coronary artery arose from a single ostium within the right sinus of Valsalva (Fig. 1A). Chest computed tomography demonstrated a retro-aortic course of the LCA (Fig. 1B). A discussion was held among the heart failure cardiology and cardiac surgery team. Because the LCA did not appear to have an inter-arterial or other malignant course, nor did it appear to have flow limitation or restriction on angiography, the decision was made to pursue transplantation. A heart team was sent to the donor facility, and the heart was accepted and later transplanted (Additional file 1: Video). Transplantation was performed using our usual implantation technique without modification, beginning with the left atrial cuff anastomosis followed by the aortic anastomosis. The single coronary ostium was widely patent upon inspection, and did not require unroofing.

The patient did well post-transplant. His hospital course was prolonged primarily due to pretransplant physical deconditioning, having been hospitalized for four months prior to transplant. On posttransplant day 36, he was discharged to a rehabilitation facility, and was discharged home 24 days later.

## Discussion

Anomalous origins of coronary arteries are infrequent phenomena within the general population, and seldomly encountered at time of heart transplantation. Because left heart catheterization is not routinely performed as part of the standard evaluation of donor grafts, unless the donor is male and older than 45 (or > 50 years for female donors) or other concerns for possible coronary artery disease exist, these findings are typically unknown prior to procurement. Several reports [2–4] have documented the use of donor heart grafts with anomalous coronary anatomy. However, in most cases, these findings are often not recognized until the time of in-person donor evaluation, or even after donor cardioectomy. In this case, left heart catheterization was performed prior to evaluation by the surgical team, and thus, the diagnosis of single coronary artery was known beforehand.

Various variations of aberrant coronary anatomy exist, all with separate risk profiles. The most lethal variant described is the LCA originating from the right coronary sinus with an inter-arterial course, which carries the highest risk of sudden or exercise-induced death, particularly in younger patients [5]. As in our case, a retro-aortic course of an aberrant coronary is generally thought to have no hemodynamic consequence [6]. Given these facts, we felt this anatomy to be a benign variation and a suitable graft for this recipient—a gentleman of increased age and with a prolonged pre-transplant hospitalization. Given the fact the patient remained hospital-dependent for more than 4 months awaiting a heart offer, the benefits of transplantation of this donor graft appeared to outweigh the risks of continued hospitalization and waiting. When evaluating these grafts, we advocate that great care be taken to ensure there is no evidence of flow limitation or restriction through the anomalous coronary artery. Any suspicion of ischemia through donor history or cause of death should be of concern. When performing angiography of these vessels, intravascular ultrasound may be a useful tool to rule out impingement, restriction, or any other flow-limiting lesions of these anomalous vessels if concern exists. Given the coronary anatomy in this case, implantation of the cardiac graft did not require modification from usual practice, unlike that of Vasseur and colleagues, who applied a slight modification using a shorter pulmonary artery trunk and longer aortic trunk to create a wide aorto-pulmonary window for implantation of a graft with an inter-arterial LCA [3].

Though a relatively uncomplicated immediate posttransplant course, it is unclear how this graft may perform in the long-term. A previous retrospective analysis [1] has suggested that perhaps anomalous coronary artery patters may demonstrate a higher prevalence of significant atherosclerosis, however, little is known about the development of chronic allograft vasculopathy within a transplanted graft .

## Conclusions

In this report, we demonstrate the successful identification and utilization of a donor cardiac graft with anomalous single coronary anatomy for heart transplantation. In select cases these grafts can be successful utilized for transplantation with excellent outcomes.

## Supplementary Information

**Additional file 1**. Assessment of 40-year-old cardiac donor revealed single coronary argery with left and right coronary arteries arising from the right sinus of Valsalva and with a retro-aortic course. Due to non-malignant course of the left coronary artery, cardiac graft was deemed suitable for transplantation.

## Data Availability

The datasets used and/or analyzed during the current study are available from the corresponding author upon reasonable request.

## Abbreviation

LCA: Left coronary artery
